# Castration for Crime Not Justifiable nor Effective

**Published:** 1896-04

**Authors:** 

**Affiliations:** Sealey, Texas


### Correspondence.

### Castration for Crime Not Justifiable nor Effective.

LETTER FROM DR. DE CAUSEY.

*Editors Texas Medical Journal:*

To subject the human race to the same laws that stockmen apply to their cattle in eliminating the poorer breeds, and castrate those who are incurably diseasesed, together with lunatics, sexual perverts, thieves and criminals, would, I think, if practicable, be retrogressive, and unworthy of the soaring aspirations of scientific researches, which can accomplish no possible good that is not humanitarian in its application.

This I say respectfully, and with great deference for the superior culture and attainments of the advocates of the procedure, and with still greater deference for their anxious solicitude for the prevention of disease and crime. But I am unable, and, I candidly admit, unwilling, to foresee any advantage that this proposed measure would give us over our present methods, which are susceptible of very great improvements.

A law that would castrate the hopelessly diseased, would be a dynamite cartridge that would merit such a fervor of popular indignation that it would explode so violently as to destroy the custodians of the temple of liberty who enacted it, if it did not shatter the temple itself, and this for reasons too obvious for discussion. The idea is revolting to the instincts of humanity.

In erotomania, if the brain center that controls sexual passions is primarily affected, castration, under certain restrictions, may be admissible, but in sexual perversions, in which this sexual center only shares the general nervous erethrysm caused by disease of other nerve centers, castration would be analogous to the extraction of the teeth of a rabid animal to cure him of his madness. We should seek more rational methods than to treat effect for cause. And it seems to me that the few *ex parte* reports of selected cases, of the beneficial effects of castration, by no means establish it as a fact, that this is the best possible treatment for the insane, generally.

As for theft, there is no evidence, that I am aware of, that a eunuch may not be a kliptomaniac.

And as for men who horrify us by the atrocity of their crimes, there is neither vindictiveness nor maudlin sentimentality in the proposition that they should be speedily put to death. The safety of society demands it, and self-preservation is the first law of nature.

A man untrameled by the restraints which honor and conscience should exercise, if incapacitated to commit one crime, can, and may, if opportunity presents, commit another. And if life imprisonment places him where his sexual passion can never be gratified, castration would be an unnecessary favor to him. But if he should be imprisoned for awhile only, and then turned loose on the community, he would, on his best behavior, be only a miserable nonenity; useless to himself, and spurned by society. Extract the fangs of a viper, and you have a loathsome and useless reptile still. It requires the faith of a grain of mustard seed to believe that castration would engender morality, or even quicken its dormant germ, if one should exist.

We could very rarely, if at all, apply the proposed mutilation to the prevention of the first crime of an individual, but after he is caught in the perpetration of crime, we can most effectually prevent his further misdeeds by a summary ostracism from our planet in any living capacity, and take to ourselves the grim consolation that we will be annoyed and disgraced by him no more forever.

Our judicial system is greatly to blame for the increase in crime. It is pretty generally regarded as a travesty on justice. Criminal processes are often too long, too uncertain, and give the criminal too many chances to escape justice; and in this respect a castration law would doubtless share all the vicissitudes of the other criminal laws.

In view of these facts, although, in principle, opposed to extra judicial combinations, I am inclined to contribute my mite for the erection of a statue in memory of Judge Lynch, on which Justice may sit with her balance until our present system is improved.

What we need is fewer and simpler laws and more rigid execution.

Sealey, Texas, March 1, 1896.

